# Advances in AI for detecting pulmonary inflammation and perioperative medicine: a mini-review

**DOI:** 10.3389/fmed.2026.1865505

**Published:** 2026-07-27

**Authors:** Kecheng Huang, Xiaoyang Liang, Rongpeng Pi, Junmin Dai, Xinping Lei, Jieyu Fang

**Affiliations:** 1 Department of Anesthesiology, Guangxi Hospital Division of The First Affiliated Hospital, Sun Yat-sen University, Nanning, Guangxi, China; 2 Department of Anesthesiology, The First Affiliated Hospital, Sun Yat-sen University, Guangzhou, Guangdong, China

**Keywords:** artificial intelligence, deep learning, perioperative medicine, pneumonia, pulmonary inflammation

## Abstract

With increasing human longevity, early recognition and treatment of pneumonia in the elderly are crucial to prevent disease progression. Artificial intelligence (AI) is rapidly transforming the detection and management of pulmonary inflammation (pneumonia, COVID-19 lung damage). Accurate preoperative assessment of pneumonia contributes to improved perioperative surgical and anesthesia management. This mini-review highlights key advances: (1) Hybrid deep learning models achieve high accuracy (>96%) in analyzing ultrasound videos for disease differentiation. (2) Self-supervised learning enables expert-level X-ray interpretation without extensive annotations. (3) Multimodal integration combines imaging (CT/X-ray) with clinical data, enhancing lesion visibility and pathogen-specific diagnosis (viral vs. bacterial AUC: 0.95). Clinically, AI demonstrates high efficacy in COVID-19 detection (AUC: 0.992), pediatric pneumonia diagnosis (89–96% accuracy), and identifying post-COVID complications. Despite this promise, challenges remain, including data bias, limited pediatric datasets, “black-box” model interpretability, and ethical concerns. Future progress depends on expanding diverse training data (e.g., via federated learning), integrating explainable AI (XAI), and ensuring equitable access. In conclusion, AI offers accurate, scalable solutions for pulmonary inflammation diagnostics, with significant potential to augment clinical decision-making and extend into proactive areas like perioperative medicine for complication screening and prevention.

## 1 Introduction

Given the advancement in human longevity, pneumonia is a highly prevalent disease in older patients. Early identification and immediate intervention are essential strategies to avoid adverse outcomes. Pneumonia is a frequent comorbidity in surgical patients before operations and a common complication during the postoperative period. Accurate preoperative assessment of a patient’s pneumonia status facilitates prognostic prediction and perioperative anesthesia management (1). The integration of artificial intelligence (AI) into medical diagnostics has brought about a revolutionary change in the detection and management of pulmonary inflammation, such as pneumonia, COVID-19-related lung damage, and other respiratory disorders (2–5). By leveraging deep learning and multimodal imaging analysis, AI systems now attain diagnostic accuracy that is comparable to or even exceeds that of human experts (6–8). The application of artificial intelligence in healthcare still faces certain limitations (9–11).

This review summarizes the recent breakthroughs in AI-driven pulmonary inflammation detection, with a focus on technological innovations, clinical applications (especially in perioperative settings), and future challenges (12–16). Advances in artificial intelligence technology hold significant potential for enhancing the assessment and treatment of perioperative pneumonia and lung function (17–20).

Search Strategy and Selection Criteria As a mini-review, a targeted literature search was conducted to identify relevant studies following fundamental principles of the PRISMA guidelines (21). We searched primary databases including PubMed, Web of Science, and IEEE Xplore for peer-reviewed articles published between January 1, 2018 and December 30, 2024. The search strategy utilized combinations of keywords such as “Artificial Intelligence,” “Deep Learning,” “Pulmonary Inflammation,” “COVID-19,” “Perioperative Medicine,” and “Intraoperative Monitoring.” Articles were selected based on their relevance to technological innovations, clinical translation, and impact on perioperative respiratory care.

Search Strategy and Selection Criteria: As a mini-review, a targeted literature search was conducted to identify relevant studies following the fundamental principles of the PRISMA guidelines (21). We searched primary databases including PubMed, Web of Science, and IEEE Xplore for peer-reviewed articles published between January 1, 2018 and December 30, 2024. The search strategy utilized combinations of keywords such as “Artificial Intelligence,” “Deep Learning,” “Pulmonary Inflammation,” “COVID-19,” “Perioperative Medicine,” and “Intraoperative Monitoring.”

To ensure the transparency and reproducibility of the included literature, we applied specific inclusion and exclusion criteria. Inclusion criteria comprised: (1) original research articles focusing on the application of AI in pulmonary inflammation or perioperative respiratory care; (2) studies reporting specific quantitative performance metrics (e.g., AUC, accuracy, sensitivity); and (3) peer-reviewed publications in English. Exclusion criteria included: (1) non-English literature; (2) conference abstracts without full texts or lacking sufficient methodological details; and (3) studies without clinical performance evaluation. To prevent the risk of double counting and potential bias, quantitative performance metrics were extracted exclusively from original scientific articles, while review articles and meta-analyses were utilized solely to discuss broader technological trends and future directions. While a comprehensive quality assessment tool was not applied due to the scope of a mini-review, a detailed PRISMA flowchart illustrating the study selection process has been provided in Supplementary Figure 1.

## 2 Technological innovations in AI models

### 2.1 Hybrid deep learning architectures

Recent studies have emphasized the success of hybrid models that combine convolutional neural networks (CNNs) and long short-term memory (LSTM) networks (22–24). For example, the TD-CNNLSTM-LungNet model, developed by Australian universities, analyzes ultrasound videos to detect lung diseases with an accuracy of 96.57% (25). This model identifies subtle pixel-level patterns in ultrasound frames through CNNs, while LSTMs contextualize temporal changes, effectively differentiating between pneumonia, COVID-19, and other conditions. Such architectures reduce false negatives (with a recall rate of 96.51%), which is crucial for time-sensitive diagnoses (26). Intraoperative pulmonary ultrasound serves as a valuable tool for the real-time assessment of dynamic pathophysiological changes in the lungs, including the detection of atelectasis, pulmonary edema, and estimation of pulmonary artery pressure (25, 27–31). By providing immediate visual feedback, it enhances precision in fluid administration and supports the implementation of proactive, lung-protective ventilation strategies at an early stage, thereby potentially improving perioperative respiratory outcomes (32–35). Nevertheless, the utility of intraoperative ultrasound remains limited in practice due to constraints imposed by the sterile operative field, surgical site accessibility, and suboptimal patient positioning. Recent advances in wearable ultrasound technology, combined with artificial intelligence for automated interpretation of real-time imaging, hold promise for broader clinical integration (36, 37).

### 2.2 Self-supervised learning for X-ray analysis

AI models trained via self-supervised learning, such as CheXNet, demonstrate expert-level performance in interpreting chest X-rays. Stanford researchers reported that CheXNet outperformed radiologists in diagnosing 14 pathologies, including pneumonia, by learning from over 100,000 annotated images (38, 39). Similarly, a Nature Biomedical Engineering study showcased an AI model achieving radiologist-level accuracy in detecting pneumonia and pulmonary failure from unannotated X-rays (40).

Artificial intelligence (AI) has made remarkable progress in the field of lung inflammation detection, becoming a key technological driving force in enhancing the efficiency and accuracy of medical imaging diagnosis. AI systems can automatically analyze chest X-rays and CT images for early screening and accurate diagnosis, identifying subtle lesions and ground-glass opacities characteristic of various pulmonary inflammations, including COVID-19 pneumonia, bacterial pneumonia, or viral pneumonia, with an accuracy rate exceeding 90%, significantly reducing missed diagnoses and misdiagnoses (41–44).

### 2.3 Intraoperative dynamic monitoring

The integration of artificial intelligence with wearable vital sign monitoring devices enables real-time, continuous tracking of key parameters—including respiratory rate, oxygen saturation, heart rate and body temperature—both during and after surgical procedures. This AI-enhanced system facilitates the automated detection of abnormal physiological fluctuations and provides early alerts, thereby supporting timely clinical intervention. Such an approach holds significant potential for reducing the incidence of postoperative pneumonia and other respiratory complications, ultimately enhancing patient safety and outcomes (45, 46).

### 2.4 Multimodal data integration

Advanced tools like DLPE (Deep Lung Parenchyma Enhancement) integrate AI with CT scans to reveal lung fibrosis and COVID-19-induced damage invisible to traditional methods. By filtering non-parenchymal features (e.g., blood vessels), DLPE enhances lesion visibility, aiding in post-COVID recovery monitoring (47). Another system, AI-ADS, combines CT and clinical data to differentiate viral pneumonia (AUC: 0.95) from bacterial cases, with segmentation accuracy (Dice coefficient: 0.85) rivaling expert radiologists (48).

Multimodal data fusion and risk assessment: The latest multimodal AI models (such as the MMI model developed by Sichuan University) not only integrate multidimensional data including imaging, medical history, and laboratory testing, but also accurately differentiate between different pathogen types, and assess the risk of critical illness, which is of great value for early warning and therapeutic decision-making for severely ill patients, and its performance is comparable to that of experienced clinicians (49, 50).

### 2.5 Automated quantitative analysis and follow-up

Artificial intelligence (AI) offers valuable support in clinical patient management through automated quantitative analysis and longitudinal follow-up. Key capabilities include the detection of radiologic abnormalities, rapid quantification of lesion volume and density, and automated comparison of imaging studies before and after intervention. Furthermore, AI-driven tools can track inflammatory changes over time, assess therapeutic response, and contribute to personalized treatment planning and follow-up care. By integrating these analytical functions, AI enhances objective decision-making and facilitates individualized patient management (51, 52).

### 2.6 Emerging AI technologies

Beyond traditional CNNs and LSTMs, the landscape of medical image analysis is being rapidly reshaped by emerging AI technologies. Vision Transformers (ViTs) have demonstrated superior capability in capturing global contextual relationships in chest imaging compared to localized CNN architectures. Furthermore, the advent of foundation models for medical imaging and Large Language Models (LLMs) is beginning to revolutionize radiology workflows. These advanced models, pre-trained on massive, unannotated multimodal datasets, can generate automated radiological reports and assist in complex clinical reasoning. Additionally, Generative AI and advanced segmentation tools like the Segment Anything Model (SAM) are being adapted for medical use, enabling rapid and zero-shot segmentation of pulmonary lesions without the need for exhaustive dataset-specific annotations. These cutting-edge tools represent the next frontier in AI-assisted pulmonary diagnostics.

## 3 Clinical applications and performance

### 3.1 COVID-19 and post-pandemic challenges

AI has been pivotal in addressing COVID-19 complications. For example, DLPE identified lung fibrosis in 60% of severe COVID-19 survivors, enabling targeted rehabilitation (53). Post-pandemic systems like AI-ADS achieved AUCs of 0.992 for COVID-19 detection and 0.958 for community-acquired pneumonia, demonstrating adaptability to emerging pathogens (54).

### 3.2 Pediatric pneumonia diagnosis

Children’s unique lung physiology (e.g., narrow bronchi, weak ciliary clearance) complicates pneumonia diagnosis (55). AI models trained on pediatric CT/X-ray datasets now achieve 89–96% accuracy in early detection, though challenges remain due to limited datasets and underrepresentation of rare pathogens (56). In the pediatric perioperative setting, AI-driven evaluation of preoperative chest imaging is highly clinically relevant. Children with undetected subclinical respiratory infections or early-stage pneumonia face a significantly higher risk of perioperative adverse respiratory events, such as laryngospasm or bronchospasm. AI tools can assist anesthesiologists in the early identification of these pulmonary conditions, thereby facilitating optimal timing for elective surgeries and allowing for customized airway management strategies to mitigate postoperative pulmonary complications.

Future efforts aim to expand pediatric-specific training data to improve subtype differentiation (57–59).

### 3.3 Pathogen-specific differentiation

Distinguishing bacterial, viral, and fungal pneumonia is critical for treatment. This strategy enables the initiation of pathogen-directed antimicrobial agents before full antibiotic susceptibility profiles become available, minimizing delays in therapy initiation. By prioritizing early targeted treatment over awaiting culture results, this approach may mitigate the risk of clinical deterioration, reduce the likelihood of progression to severe illness, and optimize the use of limited healthcare resources (41, 48, 60, 61). The TD-CNNLSTM-LungNet model excels here, using thermal maps to highlight decision-making regions, thereby improving clinician trust (25). Similarly, AI-ADS differentiates viral vs. non-viral pneumonia with 88.5% sensitivity and 91.0% specificity (62).

AI systems can complete a chest CT analysis in 20 s, automatically locate lesions, provide risk alerts, and staging recommendations, thereby significantly reducing the workload of radiologists and increasing the speed of diagnosis. These systems have been put into actual clinical use and are widely used in public health emergencies such as the COVID-19 pandemic (63–65).

### 3.4 AI applications in perioperative medicine

Although perioperative medicine shares overlapping diagnostic needs with general pulmonary care, it presents unique temporal and clinical challenges. Recent advancements have expanded AI’s role across the entire perioperative continuum. Preoperatively, AI models analyzing routine chest X-rays and electronic health records can provide robust perioperative respiratory risk prediction, identifying patients at high risk for postoperative pulmonary complications (25). Intraoperatively, integrating AI with continuous monitoring systems (e.g., capnography, pulse oximetry, and wearable sensors) supports AI-assisted anesthesia management and intraoperative monitoring, offering real-time alerts for impending hypoxemia or atelectasis (38, 39). Postoperatively, AI-based perioperative decision support systems leverage multimodal data (including intraoperative hemodynamics, fluid balance, and postoperative imaging) to predict and detect complications such as pneumonia or acute respiratory distress syndrome (ARDS) at an early stage (45, 46). While these AI-driven perioperative applications show tremendous potential to optimize patient safety, prospective clinical validation in diverse surgical cohorts remains limited and warrants further investigation (Table 1).

**Table 1:** Comprehensive summary of key AI technologies and clinical applications in pulmonary inflammation and perioperative medicine.

Reference (Author, Year)	Study design	Clinical application & target	Imaging modality/data source	Dataset size (train/test)	AI architecture	Evaluation metrics	External validation	Strengths & limitations
Abian AI et al., 2024 (25)	Prospective study	Respiratory disease diagnosis (Pneumonia, COVID-19)	Ultrasound videos	Custom dataset (size not explicitly detailed in abstract)	Hybrid (TD-CNNLSTM-LungNet)	Accuracy: 96.57%, Recall: 96.51%	No	Strengths: High temporal resolution. Limitations: Single-center, lacks prospective validation.
Lhommet C et al., 2020 (48)	Prospective cohort	Viral vs. Bacterial Pneumonia differentiation	CT scans + Clinical Data	Prospective cohort, specific size not detailed in abstract	Multimodal (AI-ADS)	AUC: 0.95, Segmentation Dice: 0.85	No (Single-center)	Strengths: High specificity, rapid analysis. Limitations: Retrospective design, potential dataset bias.
Rajpurkar P et al., 2018 (38)	Retrospective study	Pathology detection from unannotated images	Chest X-ray	>100,000 images (ChestX-ray8), Validation set: 420 images	Self-Supervised Learning (CheXNet)	Expert-level diagnostic accuracy	No (Internally validated)	Strengths: Vastly reduces annotation burden. Limitations: Inherent “black-box” nature, retrospective testing.
Zhou L et al., 2022 (47)	Retrospective study	Post-COVID lung fibrosis monitoring	CT scans	Multi-cohort COVID-19 CT scans	Deep Learning (DLPE)	Identified fibrosis in 60% of severe cases	Yes (Multi-center)	Strengths: Enhances subvisual lesion visibility. Limitations: Requires extensive computational resources.
Huang K et al., 2025 (50)	Viewpoint	Perioperative hypoxemia prediction	Lung imaging, functional analysis, blood gas metrics	Retrospective clinical cohorts	Multimodal Deep Learning	Performance comparable to experienced clinicians	No	Strengths: Integrates multidimensional data for proactive screening. Limitations: Prospective validation needed.
Mahato K et al., 2024 (45)	Review	Comprehensive health and postoperative monitoring	Hybrid multimodal wearable sensors	Various trial cohorts	AI-enhanced monitoring algorithms	Real-time dynamic detection of abnormal physiological fluctuations	Yes	Strengths: Continuous, real-time tracking of critical vitals. Limitations: Hardware integration and patient comfort challenges.

AI, artificial intelligence; CNN, convolutional neural network; LSTM, long short-term memory; AUC, area under the receiver operating characteristic curve; CT, computed tomography; EHR, electronic health record.

## 4 Challenges and future directions

### 4.1 Data limitations, bias, and overfitting risks

Most AI systems rely on datasets skewed toward common diseases (e.g., COVID-19, bacterial pneumonia), neglecting rare subtypes (66). For pediatric applications, small sample sizes (often fewer than 5,000 cases) limit the diversity and representativeness of data, thereby compromising model generalizability and performance across varied populations and settings (67). Furthermore, while many reviewed studies report exceptionally high performance metrics (e.g., accuracies >96% or AUCs >0.99), it is critical to interpret these results cautiously. High performance in retrospective, single-center studies does not necessarily translate into real-world clinical effectiveness. These metrics may reflect dataset heterogeneity, class imbalance, or severe model overfitting rather than true clinical utility. The lack of robust external validation across different institutions and populations remains a significant barrier to clinical deployment readiness. To address these limitations, solutions include federated learning and international collaborations to pool diverse data. Federated learning offer a promising pathway by enabling decentralized model training across multiple institutions without sharing raw data. Coupled with international collaborations that aggregate diverse and multisource datasets, these approaches can significantly enhance the robustness, fairness, and clinical applicability of AI models in underrepresented domains such as rare disease diagnosis and pediatric care (68). Coupled with prospective clinical evaluations to establish statistical confidence intervals, these approaches are essential to bridge the gap between technical performance and actual clinical utility.

### 4.2 Interpretability and clinician trust

While tools like thermal maps in TD-CNNLSTM-LungNet enhance transparency, many “black-box” models still face skepticism. Integrating explainable AI (XAI) frameworks, such as attention mechanisms, could bridge this gap (69). Furthermore, the lack of interpretability significantly impedes the integration of AI systems into critical clinical decision-making processes. Establishing sufficient trust and comprehensive understanding among clinicians regarding AI-assisted diagnosis of pulmonary inflammation remains a considerable challenge (70).

### 4.3 Regulatory and ethical considerations

The FDA’s approval of AI-driven devices (e.g., DermaSensor for skin cancer) sets precedents for pulmonary tools (71). However, ethical concerns such as over-reliance on AI and data privacy require rigorous governance, especially in low-resource settings (72).

Techniques ranging from hybrid models analyzing ultrasound videos and multimodal data streams (e.g., respiratory waveforms, oxygenation trends, hemodynamic parameters, and imaging) to self-supervised X-ray systems exemplify AI’s capacity to augment clinical decision-making by complementing, rather than replacing, clinician expertise (19, 73). Future advancements hinge on addressing perioperative-specific data gaps, enhancing model interpretability for actionable clinical insights, and ensuring equitable access to these technologies (20). The integration of artificial intelligence (AI) into clinical practice has introduced complex challenges regarding liability attribution. The adoption of AI-driven clinical decision support systems (AI-CDSS) poses fundamental questions concerning causal, ethical, and legal accountability. While these systems can enhance the efficiency of medical care, they also obscure traditional boundaries of responsibility, potentially creating accountability gaps (74). Furthermore, the opacity of AI algorithms may render their decision-making processes difficult to interpret, complicating the assessment of whether an AI-derived decision was appropriate (75). The integration of AI systems has the potential to reshape the physician–patient relationship and introduces ethical challenges that warrant careful consideration (76).

## 5 Conclusion

Artificial intelligence (AI) is fundamentally transforming diagnostic approaches, initially demonstrated in pulmonary inflammation with rapid, accurate, and scalable solutions. This potential extends powerfully into perioperative medicine, where AI-driven analysis of multimodal data streams offers significant promise for the proactive screening and early detection of perioperative hypoxemia. By facilitating real-time risk stratification and alerting clinicians to subtle physiological derangements, AI can enhance vigilance, optimize intervention timing, and ultimately improve patient safety during the critical perioperative period.

As AI continues to evolve, its role in predicting, preventing, and managing critical respiratory complications like hypoxemia will undoubtedly expand, significantly contributing to enhanced perioperative outcomes and patient safety on a global scale.
